# Bronchiectasis—Could Immunonutrition Have a Role to Play in Future Management?

**DOI:** 10.3389/fnut.2021.652410

**Published:** 2021-04-29

**Authors:** Emma J. Derbyshire, Philip C. Calder

**Affiliations:** ^1^Nutritional Insight, London, United Kingdom; ^2^School of Human Development and Health, Faculty of Medicine, University of Southampton, Southampton, United Kingdom; ^3^National Institute for Health Research (NIHR) Southampton Biomedical Research Centre, University Hospital Southampton National Health Service (NHS) Foundation Trust, University of Southampton, Southampton, United Kingdom

**Keywords:** bronchiectasis, lung health, respiratory tract infections, immunonutrition, inflammation

## Abstract

Bronchiectasis is a chronic condition in which areas of the bronchial tubes become permanently widened predisposing the lungs to infection. Bronchiectasis is an age-associated disease with the highest prevalence in people older than 75 years. While the prevalence of bronchiectasis is higher in males, disease is more severe in females who have a poorer prognosis. The overall prevalence of the disease is thought to be rising. Its aetiology is multi-faceted, but a compromised immune system is now thought to play a central role in the pathology of this disease. Research has begun to study the role of malnutrition and certain nutrients—vitamin D and zinc—along with the role of the lung microbiome in relation to the management of bronchiectasis. Given this, the present mini review sets out to provide an overview of the state-of-the-art within the field, identify research gaps and pave the way for future developments and research investment within this field.

## Introduction

For a long time, bronchiectasis has been regarded as an “orphan disease,” a disease so rare that it was not considered commercially viable to develop drugs to treat it (1). This led bronchiectasis to be regarded as unimportant and it was subsequently under-researched (2, 3). Now, into the twenty-first century, bronchiectasis is becoming an increasingly frequent prognosis and it has even been coined as an emerging global epidemic (4). In the United Kingdom (UK) there is evidence to suggest that both the prevalence and incidence of bronchiectasis are rising quickly (5). For example, for non-cystic fibrosis bronchiectasis, the UK incidence for women rose from 21.2 per 100,000 person-years in 2004 to 35.2 per 100,000 person-years in 2013 and for men from 18.2 per 100,000 person-years in 2004 to 26.9 per 100,000 person-years by 2013 (5). It was found that bronchiectasis is more common amongst those with higher socioeconomic status, although the reason for this is unclear (5). The British Lung Foundation's “*Respiratory Health of the Nation*” project estimated that ~212,000 people in the UK are living with bronchiectasis – levels higher than previously thought, with the severity of the condition more prevalent in females, in those aged over 70 years and in the least deprived sectors of the population, which is in contrast to other respiratory disorders (6). Sex differences, with females portending to worse clinical outcomes has, in part, been explained by differences in sex steroid hormones which may affect airway microbiology (7).

Bronchiectasis is characteristically caused by abnormal dilation of the bronchi and a damaged epithelium which, in turn, impairs mucus clearance and facilitates bacterial infection (8, 9). The condition is frequently accompanied by inflammation with symptoms manifesting as a chronic cough, sputum production and recurrent infections (10). For most people the causes behind bronchiectasis are “idiopathic” but some pathophysiological mechanisms include: dysregulation of the immune system, inflammatory conditions (e.g., bowel disease or arthritis), or structural lung problems (11). Amongst the array of causative factors behind bronchiectasis, the majority of these compromise the immune response in some form resulting in an impaired ability to fight infection (12).

Treatment strategies for bronchiectasis focus primarily on improving quality of life, preventing the frequency of flare ups and offsetting disease progression (8). Long term use of antibiotics—continuous or cyclical (during the winter months)—is typically recommended to stabilise the disease (13). Increasingly, there has been a growing body of evidence linking nutrition to immune function and protection against respiratory infections (14–16). Consequently, so-called immunonutrition could also have a role in bronchiectasis management – a field of science that appears to be underexplored. This mini-review considers the science linking nutrition to bronchiectasis management and describes how this could be further advanced in the future. The focus of the present review is on non-cystic fibrosis bronchiectasis.

## Immune Dysfunction in Bronchiectasis

King et al. stated that “*the deficiency of the host immune response to bacterial infection is regarded as a primary requirement for the development of bronchiectasis*” (12). While a spectrum of causative factors underpin bronchiectasis, the majority of these compromise immune function and the ability to fight infection (12). Both the adaptive and innate immune responses are activated in bronchiectasis (12), with neutrophils having a central role in bronchiectasis pathology. Inflammation is driven by neutrophils that migrate to the airway in response to elevated concentrations of chemokines and pro-inflammatory cytokines, which are elevated in the airways of these patients (17). Neutrophils would typically phagocytose and kill pathogenic bacteria, but in patients with bronchiectasis these appear to have been disabled, possibly by the cleavage of phagocytic receptors by neutrophil elastase (17). An excessive release of neutrophil elastase is known to have damaging effects on the lungs, including impaired ciliary beat frequency, airway epithelia destruction, mucus gland stimulation and increased sputum production (18). Other mechanisms related to immune dysfunction are also evident in patients with bronchiectasis. For example, proteases produced in the lung as a result of the inflammatory response result in pathological dilation that is characteristic of bronchiectasis (12).

## Specific Immunonutrients and Bronchiectasis

Optimal functioning of the immune system is central to human health, and nutrition is one of the main exogenous factors involved in the modulation of immune function (19). The British Thoracic Society presently provides guidelines for adults with bronchiectasis, which include advice on airway clearance techniques, use of mucoactive treatments, anti-inflammatory therapies, and antibiotic use (20). Nutritional advice is not presently included, perhaps because of an historic lack of relevant evidence. However, recent research has begun to investigate the role of nutrition in bronchiectasis and respiratory infection management.

It is now becoming relatively well-established that vitamin D supplementation could help to attenuate respiratory tract infections (RTIs) (21, 22). Vitamin D receptors are present in almost all cells of the immune system, vitamin D thus being important to the regulation of both innate and adaptive immunity (23). For example, vitamin D receptors are found on monocytes which differentiate into macrophages under the effects of 1,25(OH)_2_D (24). Macrophages and dendritic cells also express their own 1α-hydroxylase isoenzyme which synthesises 1,25(OH)_2_D intracellularly (25). Vitamin D receptors are further present on T-cells, B-cells and monocytes (26). As well as possessing immunomodulatory functions, vitamin D is also known to increase the antiviral responses of respiratory epithelial cells during infections (27). Vitamin D has been found to enhance neutrophil killing in patients with bacterial respiratory tract infections (RTIs) and to lower pro-inflammatory cytokine production induced by infected neutrophils (28). Amongst patients with cystic fibrosis, vitamin D status correlated with immunoglobulin concentrations and reduced CD279 programmed death 1 (PD-1) expression on CD8^+^ T cells and the frequency of CD8^+^ T cells co-expressing CD38 and human leucocyte antigen D-related (29). Vitamin D also induces antimicrobial peptide gene expression including that of cathelicidins and defensins which possess the ability to disrupt the integrity of the pathogens, resulting in their apoptosis (30). These mechanisms could, in part, explain some of the “antibiotic” actions of vitamin D (30). Together, these observations suggest that vitamin D could have a potential extended role to play in bronchiectasis management.

Clearly, the pathogenesis of bronchiectasis is multi-faceted with autoimmune disease, conditions of immune dysregulation, genetics, geography, and ethnicity having underpinning roles (9). As shown in Table 1 several nutritional risk factors could also contribute to bronchiectasis pathology. These includes malnutrition, pro-inflammatory diets, suboptimal hydration status and vitamin D and zinc shortfalls. Malnutrition and an imbalance between energy supply and requirements can result in a negative balance between protein synthesis and breakdown, potentially affecting the prognosis of respiratory diseases (31). A growing body of evidence now links pro-inflammatory diets to disease-related fatigue whilst balanced diets comprised of whole-grains, polyphenols-rich vegetables and foods providing omega-3 fatty acids may attenuate fatigue related to chronic disease (33). Adequate hydration is important for mucociliary clearance, helping to reduce concentrations and cohesion (38).

**Table 1:** Bronchiectasis: potential modifiable nutritional risk factors.

**Dietary component**	**Explanation and potential pathway(s)**	**Reference(s)**
Malnutrition	Malnutrition (lack of adequate nutrition) can cause diverse alterations in the innate and adaptive immune responses. These include the involution of the thymus, reducing T cell numbers and responses and exacerbating the susceptibility to infections.	(31, 32)
Pro-inflammatory diets	Several foods and dietary patterns have been identified as pro-inflammatory, inducing secretion of inflammatory mediators, and free radicals. Those most studied include hydrogenated fats, processed foods, and refined sugars. In contrast, balanced diets comprised of whole-grains, polyphenol-rich vegetables and foods providing omega-3 fatty acids may attenuate inflammation (so reducing fatigue related to chronic disease).	(32–34)
Vitamin D	There is evidence from RCTs for protective effects of vitamin D on tuberculosis and likely evidence for protective effects on acute airway infection. As vitamin D deficiency is prevalent, daily oral vitamin D intake, e.g., 25 μg (1,000 IU) could be an inexpensive measure to ensure adequate status in at-risk groups. Vitamin D interacts with antigen presenting cells and T-lymphocytes to promote and to regulate different stages of immune response. It has also been found to modulate antimicrobial peptide pathways and induce immune activation in patients with cystic fibrosis.	(29, 30, 35, 36)
Zinc	Zinc is regarded as the gatekeeper of immune function and could be of benefit in instances when the immune system is functioning poorly. Zinc is thought to activate key signalling molecules and induce epigenetic modifications that underpin its roles as a gatekeeper of immune function.	(37)
Water	Rehydration could potentially help to reduce mucus concentration and cohesion. EFSA advise water intakes of 2 litres/day for females and 2.5 litres/day for males from food and beverage sources.	(38, 39)

Several trials suggesting a link between low vitamin D status and bronchiectasis have been published (Table 2). Scottish research measuring serum vitamin D levels in patients with bronchiectasis (*n* = 402) found that 50% were deficient, 43% insufficient and only 7% had sufficient status (46). Additionally, 24% of the vitamin D deficient patients were colonised with *Pseudomonas aeruginosa*, had elevated sputum inflammatory marker levels and were more likely to have a decline in lung function at follow-up (46). A case-control study involving 130 Turkish cases with bronchiectasis observed that vitamin D deficiency was common and significantly associated with reduced forced vital lung capacity (40). Similarly, a pilot study conducted in New Zealand found that adults with bronchiectasis had a tendency towards sub-optimal serum vitamin D levels and weekly supplementation with 0.6 mg vitamin D3 (after a loading dose of 2.5 mg) significantly increased serum vitamin D levels (43). Amongst patients with primary ciliary dyskinesia (64% with bronchiectasis) hypovitaminosis D was evident in 79% (45). Interestingly, in a recent case study of a young male with severe acute respiratory syndrome coronavirus-2 (SARS-CoV-2), the virus which causes COVID-19, bronchiectasis and *Bordetella* bronchiseptica colonisation were treated successfully with a vitamin D3 intervention, doxycycline and intensive care (41).

**Table 2:** Summary of studies of nutrients and the lung microbiota in bronchiectasis.

**References**	**Type of study**	**Point of focus**	**Main findings**
Niksarlioglu et al. (40)	Case-Control	Vitamin D	Vitamin D deficiency was common in patients with bronchiectasis and associated with significantly lower forced vital capacity.
Fagihi et al. (41)	Case Study	Vitamin D	Co-infection of SARS-CoV-2 and *Bordetella bronchiseptica* in a bronchiectasis case was treated with vitamin D supplementation (100,000 IU bolus dose and weekly dose of 25,000 IU), doxycycline and standard intensive care.
Woo et al. (42)	Observational	Lung microbiota	The lung microbiota of patients with non-cystic fibrosis bronchiectasis was relatively stable.
Bartley et al. (43)	Pilot Intervention Study	Vitamin D	Adults with bronchiectasis had higher than expected serum vitamin D levels and standard oral vitamin D3 supplementation further increased this and health-related quality of life measures.
Byun et al. (44)	Cross-sectional	Lung microbiota	Lung microbiota was no different between stable and exacerbated bronchiectasis.
Mirra et al. (45)	Pilot Cross-sectional study	Vitamin D	79% of patients with bronchiectasis had vitamin D deficiency-to-insufficiency; hypovitaminosis D was common.
Chalmers et al. (46)	Observational Study	Vitamin D	Vitamin-D deficiency was common in bronchiectasis and correlated with bacterial colonisation, inflammatory mediator levels in sputum and reductions in lung function.
Javadmoosavi et al. (47)	Cross-sectional study	Zinc	Serum zinc levels were lower in patients with bronchiectasis compared with controls.
Tunney et al. (48)	Cross-sectional	Lung microbiota	After treatment of patients with bronchiectasis for exacerbations (flare ups) no changes in lung microbiota composition were observed.

Zinc is another nutrient known to support the function of many immune cell types and to benefit respiratory infections (49). Zinc is the chief regulator of signal transduction and cellular function, with zinc deficiency altering zinc homeostasis and molecular mechanisms including the actions of kinases, caspases, phosphatases, and phosphodiesterases which can exacerbate infection susceptibility (50). *In vivo* research also shows that zinc deficiency modulates the function and number of monocytes, neutrophils, natural killer, B-, and T-cells, with T-cell balance and function being particularly receptive to changes in zinc status (51).

To date only one study has investigated a potential role of zinc in bronchiectasis; the study found that serum zinc levels were significantly lower in patients with bronchiectasis compared with controls (47). The authors concluded that zinc could have extended benefits helping to modulate the immune system in these patients (47). Therefore, studies are now warranted that focus on zinc status/supplementation programmes in relation to specific markers of immune function (e.g., T-cell function), lung function and infection in bronchiectasis cases.

The microbiota also interacts with the local immune system. Most research has focussed on the role of the gut microbiota, which may affect both gastrointestinal and respiratory illness, but the lung microbiota is also now recognised to be important (52, 53). The lung airway microbiota is low in biomass compared to the gut, yet could be a powerful regulator of the immune system (54). As with the gut there is likely to be a bidirectional relationship between the lung microbiota and host epithelial and immune cells. For example, lung epithelial cells are being recognised as active effectors of microbial defence, contributing to both adaptive and innate immune function in the lower respiratory tract (55).

A small number of studies have begun to investigate the profile of the lung microbiota in patients with bronchiectasis (42, 44, 48). In general, the lung microbiota has been found to be relatively stable in these patients, with few differences between remission and flare-up of disease although comparisons with healthy adults are warranted (42, 44, 48).

## Discussion

Immunonutrition could be a means of reducing bronchiectasis-related infection and disease progression, which over time can result in impaired lung function. A number of nutrients support the immune system including vitamins B6, B9 (folate), B12, C, D and E, zinc, copper, iron and selenium and essential amino acids and fatty acids (15, 16, 56). There is now meta-analytical evidence available, which demonstrates that vitamin D supplementation reduces the risk of respiratory tract infections (21, 22, 57). In the UK, Biobank data shows that Asian and black residents have higher rates of vitamin D deficiency (54 and 35%) than Caucasians (12%) and socioeconomic deprivation, smoking and northerly latitudes increased the odds of vitamin D deficiency (58).

Zinc may also reduce risk of such infections and a recent rapid review of 118 publications concluded that zinc could reduce the risk, duration, and severity of SARS-CoV-2 and other respiratory virus infections through several mechanisms which include its potential to reduce inflammation, interfere with SARS-CoV-2 replication, improve mucocilliary clearance, promote antiviral immunity and reduce the risk of co-infection with pneumonia (59). Malabsorption syndromes and the consumption of high phytate-containing cereals in the developing world have been associated with high rates of zinc deficiencies (60). In addition to traditional essential nutrients, many plant-derived dietary components, including flavonoids, may also support the immune response and regulate inflammation. A meta-analysis of 14 studies reports that flavonoid supplementation reduced upper respiratory tract infection incidence by 33% compared with control, although differences between immune markers between groups were found to be modest (61).

As indicated earlier, there are observations of low vitamin D and zinc status being linked to bronchiectasis, but randomised controlled trials looking at effects on immune markers, lung function and infection with these two nutrients, and with other potential candidates, are lacking. This is an important research gap. Currently neither the British Thoracic Society nor the European Respiratory Society bronchiectasis guidelines include any nutritional advice (20, 62). Given the potential for nutrients to play a useful role in disease management, more research in this area is warranted. Current advice for the UK general population is to take a 10 microgram daily supplement of vitamin D to keep bones, teeth, and muscles healthy (63). However, intakes of 2,000 IU/day (50 micrograms/day) seem to be needed to reduce the risk of respiratory tract infections (16). Vitamin D deficiency may play a role in the pathogenesis of bronchiectasis and vitamin D supplementation could be a cost-effective therapeutic approach to help manage and attenuate the severity and progression of this disease (64), if it was supported by quality research evidence.

Regarding the roles of other nutrients and the lung microbiota and its modulation, there is presently not enough evidence to make any clear conclusions. Research on candidate nutrients other than vitamin D is warranted; high quality trials focusing on vitamin D, zinc, other nutrients, and flavonoids and immune- and bronchiectasis-markers would be worthy of future study (15, 16, 65). Research focusing on the gut and lung microbiota in patients with bronchiectasis over extended time periods and using larger sample sizes to capture disease heterogeneity during stability and at exacerbation is warranted (66).

## Concluding Remarks

Bronchiectasis is a progressive immune-infective-inflammatory airway condition that results in a vicious cycle of repeated exacerbations and irreversible damage. It is becoming more common. Whilst conventional medical approaches are clearly crucial to its management, immunonutrition could also have a viable role to play, with evidence for vitamin D currently being the most promising. Given growing interest in lung health, in part sparked by the SARS-CoV-2 pandemic, greater focus and investment in research is needed in this disease.

## Author Contributions

ED and PC compiled, researched, wrote, and edited the review. All authors contributed to the article and approved the submitted version.

## Conflict of Interest

ED is the founder of Nutritional Insight. The remaining author declares that the research was conducted in the absence of any commercial or financial relationships that could be construed as a potential conflict of interest.
